# Validation of the English version of the Mood Rhythm Instrument

**DOI:** 10.1186/s40359-020-00397-2

**Published:** 2020-04-17

**Authors:** Melissa A. B. Oliveira, Kristina Epifano, Salina Mathur, Felipe Gutiérrez Carvalho, Marina Scop, Alicia Carissimi, Ana Paula Francisco, Luciene L. S. Garay, Ana Adan, Maria Paz Hidalgo, Benicio N. Frey

**Affiliations:** 1 Laboratório de Cronobiologia e Sono do Hospital de Clínicas de Porto Alegre (HCPA), Universidade Federal do Rio Grande do Sul (UFRGS), Porto Alegre, Rio Grande do Sul Brazil; 2 Programa de Pós-Graduação em Psiquiatria e Ciências do Comportamento – Faculdade de Medicina, UFRGS, Porto Alegre, Rio Grande do Sul Brazil; 3 Department of Psychology, Neuroscience & Behaviour, McMaster University, Hamilton, ON Canada; 4 Mood Disorders Program and Women’s Health Concerns Clinic, St. Joseph’s Healthcare, Hamilton, ON Canada; 5 Department of Clinical Psychology and Psychobiology, School of Psychology, University of Barcelona, Barcelona, Spain; 6 Institute of Neurosciences, University of Barcelona, Barcelona, Spain; 7 Department of Psychiatry and Behavioural Neurosciences, McMaster University, 100 West 5th Street, Suite C124, Hamilton, ON L8N 3K7 Canada

**Keywords:** Circadian rhythm, Chronobiology, Sleep, Depression, Mood

## Abstract

**Background:**

Disruption of biological rhythms has been linked to the pathophysiology of mental disorders. However, little is known regarding the rhythmicity of mood symptoms due to the lack of validated clinical questionnaires. A better understanding of the rhythmicity of mood symptoms can help identifying individuals whose severity of mood symptoms follows an altered circadian rhythm. The objective of this study was to validate the English version of the Mood Rhythm Instrument (MRhI), a self-reported measure of self-perceived rhythmicity of mood symptoms and behaviours, in a sample of the general population from Canada.

**Methods:**

After the translation process, the final English version of the Mood Rhythm Instrument (MRhI-English) was applied on participants recruited at McMaster University and St. Joseph’s Healthcare Hamilton campuses. Individuals were also asked to answer the Reduced Morningness-Eveningness Questionnaire (rMEQ).

**Results:**

Four hundred one individuals completed the English version of the MRhI and the rMEQ. The MRhI-English presented a Cronbach’s alpha of 0.75. The factorial analysis grouped the MRhI-15 items in 3 factors (cognitive, affective and somatic), with affective items having a lower frequency of self-reported 24-h peaks. Comparison between sexes showed that women reported a higher frequency of daily peaks in *irritability*, *anxiety*, *sadness* and *talking to friends*, while men exhibited peaks more frequently in *problem-solving*, *sexual arousal* and *motivation to exercise*.

**Conclusions:**

Our findings suggest that the English version of the MRhI displayed good internal consistency. Future directions will include the use of the MRhI instrument in individuals with mood disorders, aiming to provide a better understanding of the relationship between daily patterns of mood variability and mental health outcomes.

## Background

Mood disorders are chronic mental health conditions that cause a range of disabilities for patients, generating a negative impact on the individual, health systems and society [1]. Due to their multifactorial etiology, mood disorders are known to be influenced by genetic, personal and/or environmental factors [2, 3]. While mood disorders are prevalent, a significant proportion of individuals with mood disorders go undiagnosed due to the spectrum of severity and prognosis [4, 5]. In this context, it is important to find ways to improve the identification of its risk factors, leading to appropriate treatment management and consequently preventing unfavourable outcomes.

The etiology of mood disorders has been extensively studied, and some chronobiological factors have been found to play an essential role in the pathophysiology of mood disorders [6]. For instance, previous studies have revealed that alterations in circadian rhythms are highly associated with major depression and bipolar disorder [7–9]. Specifically, altered variations of the clock genes, those involved with rhythmicity and timing of biological rhythms at a molecular level, have been found in individuals with bipolar disorder, as well as major depression [10]. Recent meta-analytic studies have concluded that the abnormal sleep rhythms are consistently observed in patients with these two major mood disorders [11, 12]. These disturbances can be a potential predictor of declining mental health, as they have been shown to contribute to escalated mood levels and the triggering of manic episodes in patients [13]. For instance, studies have shown that sleep deprivation and jet lag can trigger or aggravate depressive, hypomanic or manic episodes [14]. In addition, studies have shown that discrete patterns of daily activity rhythms can distinguish specific mood disorder subgroups, such as bipolar depression and mania, or non-melancholic and melancholic depression [15, 16]. Notably, lower stability and weakened amplitude in rest-activity rhythms have been associated with greater symptom severity (e.g. impulsivity and mood instability) in individuals with borderline personality disorder [17].

Therapies that target circadian rhythms synchronization might be useful in the management of mood disorders, such as bright light therapy and interpersonal and social rhythm therapy [18–20]. A better understanding of the rhythmicity of mood symptoms can help to identify individuals whose severity of mood symptoms follow an altered circadian rhythm. However, despite the increasing evidence linking mood disorders and circadian rhythms disruption, little is known regarding the rhythmicity of mood symptoms due to the lack of validated clinical questionnaires. In order to fill this gap, we have developed the Mood Rhythm Instrument (MRhI), a clinical tool aiming at assessing the self-perceived rhythmicity of mood symptoms.

The MRhI is a self-reported questionnaire developed to evaluate the presence and timing of daily patterns for mood-related symptoms over the last 15 days. Each of the 15 items comprises a categorical and a continuous question. The original version of this instrument was created in Brazilian Portuguese [21], which was then translated and validated in Spanish [22, 23]. In a large study with 708 participants that completed the MRhI, we found that the rhythmicity of specific mood-related symptoms and behaviors, such as pessimism and motivation to exercise were associated with higher risk for psychiatric disorders [24]. Notably, we also found specific cultural differences in comparing Spanish and Brazilian samples in terms of the daily patterns of mood-related symptoms [25]. These results are consistent with previous studies suggesting that cultural differences, as seen in different populations’ sleep/wake habits [26–28], as well as ethnic differences [29], and as racial differences in tau and circadian phase shifting, are relevant factors in circadian rhythm research. Thus, this study aims to validate the MRhI English version in an English-speaking Canadian sample.

## Methods

### Step 1. Translation of the Mood Rhythm Instrument (MRhI)

**Fig. 1:**
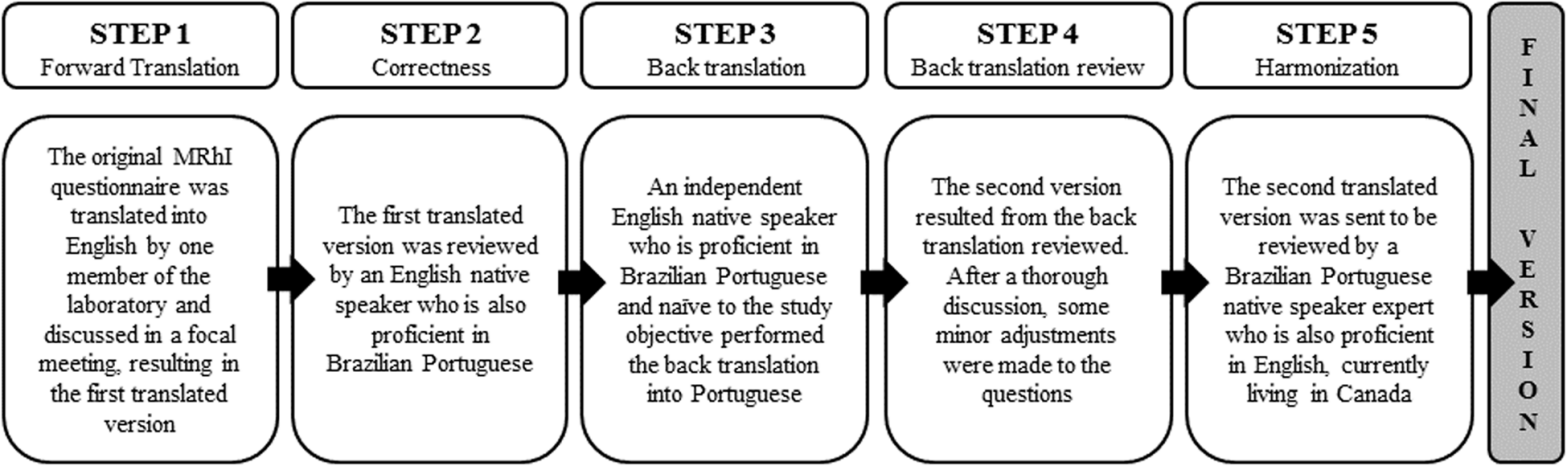
Translation process of MRhI into English. The main steps for translation and cultural adaptation of Brazilian Portuguese into English are detailed

The translation process is detailed in Fig. 1 and was composed by five steps, including forward translation, correctness, back translation, back translation review and harmonization.

The instructions on how to answer the questionnaire was updated with the removal of one sentence. In the translated version, the following sentence was written: “Answer the following questions according to the previous 15 days, taking into account how you have felt during most of the time, on the majority of the days and in the absence of any events that have caused you distress”. In order to improve clarity, this sentence was changed to: “Answer the following questions according to the previous 15 days, taking into account how you have felt during most of the time”. All sentences had their conjugation changed to the Present Perfect Tense, as the MRhI intends to assess the self-perceived rhythmicity of mood-related symptoms in the last 15 days. For question 11, the word “prone” was changed to “motivated”. For question 12, the sentence “you memorize” was changed to “your memory”. For question 15, the sentence “when you feel your best” was changed to “when you have had more energy and motivation to do things”. Furthermore, instead of a 24-h format scale as it stands in the Portuguese version, the English version has an am/pm format scale. The final English version of the MRhI can be found as Supplementary Methods.

### Step 2. Validation of the Mood Rhythm Instrument

#### Participants and procedures

Data collection was conducted between January 2016 and September 2018. We recruited the study sample through poster advertisements at McMaster University and St. Joseph’s Healthcare Hamilton campuses, and online research recruitment within the Department of Psychology, Neuroscience & Behaviour at McMaster University. The final study sample comprised 401 individuals (age: 18–60; mean age: 22. ±7), predominantly women (72%), with a mean of 15 ± 3 years of schooling. The study was approved by the Hamilton Integrated Research Ethics Board and was conducted in accordance with the Declaration of Helsinki. All study participants provided written informed consent before study entry.

#### Instruments

##### Mood Rhythm Instrument (MRhI)

The MRhI is composed of 15 items referring to physical, psychological and behavioural aspects related to mood, and each item provides a categorical (presence or absence of a daily peak) and a continuous (peak time in a 24 h period) variable. Subjects answered if, in the last 15 days, there was a specific time of the day when they experienced a peak in mood-related symptoms. We have recently completed a study where we tested the agreement rates between the MRhI and a daily version of the MRhI, and we found high agreement rates between the two instruments, thus suggesting that the MRhI may not be significantly influenced by memory bias [25]. The MRhI displayed a satisfactory internal consistency (i.e. Cronbach’s alpha) in Brazilian (0.73) and Spanish (0.70) populations [21, 23].

##### Reduced Morningness-Eveningness questionnaire (rMEQ)

The rMEQ provides a self-evaluation of chronotype, which is a unidimensional construct and offers a classification that varies between evening and morning types. This questionnaire was developed by Adan and Almirall [30] and includes items 1, 7, 10, 18, and 19 of the original MEQ. These 5 questions comprise the smallest possible number of items that provides the maximum amount of information relative to the time when each individual feel to be more prone to perform daily activities and to sleep. Higher numbers indicate morning tendencies and lower numbers indicate evening tendencies (scores 4–11: evening type; 12–17: intermediate type; 18–25: morning type). The rMEQ has been widely used due to its practicality, allowing parallel recording of other variables, especially in large sample studies. The psychometric properties of the rMEQ has been evaluated in many countries of Europe, America, as well as in Kingdom of Saudi Arabia, China, India, Iraq and Iran. In most of these previous studies rMEQ showed similar values for internal consistency [31].

#### Reliability and validity process

Internal consistency was measured with Cronbach’s alpha. A Cronbach’s alpha value between 0.7 and 0.9 was considered acceptable [32]. Psychometric properties of MRhI-English were assessed through the exploratory factor analysis (EFA). The EFA was carried out using a tetrachoric correlation matrix since our data has a binary feature [33]. Maximum Likelihood and Varimax were the extraction and rotation methods, respectively. Compared to other commonly used extraction methods, Maximum Likelihood uses the full information solution of the 2p contingency table [34]. For practical implications, Maximum Likelihood is considered preferable for tests with few factors (stated as 1 to 3 factors), which is the main reason we opted for this specific extraction method [35]. Satorra-Bentler corrected model estimation algorithm was used to surmount biased estimates. Varimax is a widely used rotation technique, being suitable for the present data as it shows excellent results by differentiating groups in several simulation scenarios [36]. Factors extraction was initially performed through Velicer’s minimum average partial (MAP) [37], Horn’s Parallel Analysis (PA) [38], and Comparison Data (CD) [39], obtaining 2, 5, and 3 factors respectively. As supported by Ruscio and Roche [39], CD method performs better than MAP and PA in terms of accuracy and precision, with nearly unbiased results. Thus, the three-factor model was considered for the analysis. A confirmatory factor analysis (CFA) using Comparative Fit Index (CFI) > 0.95, Tucker-Lewis Index (TLI) > 0.95, Root mean square error of approximation (RMSEA) < 0.06, and Standardized Root Mean Square Residual (SRMR) < 0.08 as model fit indices were conducted [40]. The CFA model fit indices showed suitable or slightly less than the good fit values (χ^2^ = 178.8, df = 87, CFI = 0.875, TLI = 0.850, RMSEA = 0.05, SRMR = 0.06).

#### Statistical analysis

Variables were tested for normality by the Shapiro-Wilk test. Comparisons of the frequency of the dichotomous MRhI-English according to sex were analyzed by Chi-square test (χ^2^). Linear-circular correlations between time peaks of MRhI items and MEQ scores were performed [41]. The distribution of MRhI-English items peaks were shown as a circular mean and compared between sexes according to Mardia-Watson-Wheeler test, considering that the data do not follow a normal distribution for circular data [42]. R version 3.4.1 (package “Directional v3.3”) and NCSS 12.0.9 were used for circular analysis. R version 3.4.1 (packages “psych”, “lavaan” and “RGenData”) and PASW Statistics Version 18 (SPSS Inc., Chicago, IL) were used for statistical analyses. Statistical significance was accepted at *p* < 0.05.

## Results

### Reliability and validity of the English version of the mood rhythm instrument

The MRhI-English presented a Cronbach’s alpha of 0.75 in this sample, which suggests good internal consistency. Table 1 presents the three factors obtained with the factorial analysis of the categorical MRhI items. The first factor was predominantly composed by cognitive items such as *problem-solving, concentration, memory, talking to friends* and *energy.* Items related to affective aspects were in the second factor, e.g. *self-esteem, anxiety, sadness* and *pessimism.* Finally, the third factor grouped *alertness, sleepiness, irritability* and somatic items like *appetite*, *sexual arousal,* and *motivation to exercise*.

**Table 1:** Exploratory Factor Analysis of the English version of the Mood Rhythm Instrument items based on a three-factor solution

Items	Factor 1	Factor 2	Factor 3
1. Alertness	0.43	0.16	**0.45**
2. Sleepiness	0.26	0.2	**0.72**
3. Problem-Solving	**0.76**	0.09	0.28
4. Self-esteem	0.15	**0.45**	0.44
5. Concentration	**0.64**	0.1	0.31
6. Appetite	0.12	0.19	**0.54**
7. Sexual Arousal	0.11	0.26	**0.45**
8. Irritability	0.15	0.37	**0.49**
9. Anxiety	0.1	**0.66**	0.21
10. Sadness	0.1	**0.85**	0.01
11. Motivation to Exercise	0.2	−0.12	**0.45**
12. Memory	**0.72**	0.24	0
13. Pessimism	0.18	**0.63**	0.14
14. Talking to Friends	**0.32**	0.31	0.17
15. Energy	**0.59**	0.11	0.46
Eigenvalues	2.38	2.26	2.32
% of variance	0.16	0.15	0.15

The frequency of self-reported rhythmicity for each MRhI item and the comparison between sexes is shown on Table 2. Items with the highest reported occurrence (> 70%) of a daily peak were *alertness*, *sleepiness*, *concentration*, *appetite* and *energy*. On the other hand, less than 40% of subjects reported a daily peak in *self-esteem*, *sexual arousal*, *sadness*, *memory* and *pessimism*. The comparison between sexes showed that women reported a higher frequency of daily patterns in *irritability*, *anxiety*, *sadness* and *talking to friends*, while men reported in *problem-solving*, *sexual arousal* and *motivation to exercise*.

**Table 2:** Frequency of self-reported rhythmicity of the Mood Rhythm Instrument (MRhI) items – English version

MRhI items	Total (*n* = 401)	Men (*n* = 112)	Women (*n* = 289)	χ^2^, *p* value
	n (%)	n (%)	n (%)	
Alertness	313 (78)	85 (76)	228 (79)	0.42, *p* = 0.52
Sleepiness	375 (94)	109 (97)	266 (92)	3.71, *p* = 0.05
Problem-solving	273 (68)	85 (76)	188 (65)	**4.36,*p*** < 0.05*
Self-esteem	144 (36)	40 (36)	104 (36)	0.00, *p* = 0.96
Concentration	318 (79)	86 (77)	232 (80)	0.60, *p* = 0.44
Appetite	292 (73)	82 (73)	210 (73)	0.01, *p* = 0.91
Sexual Arousal	125 (31)	47 (42)	78 (27)	**8.44,*p*** < 0.01**
Irritability	243 (61)	58 (52)	185 (64)	**5.06,*p*** < 0.05*
Anxiety	172 (43)	34 (30)	138 (48)	**9.97,*p*** < 0.01**
Sadness	157 (39)	30 (27)	127 (44)	**9.98,*p*** = 0.01**
Motivation to exercise	248 (62)	79 (70)	169 (58)	**4.97,*p*** = 0.05*
Memory	127 (32)	37 (33)	90 (31)	0.13, *p* = 0.72
Pessimism	122 (30)	30 (27)	92 (32)	0.97, *p* = 0.32
Talking to Friends	205 (51)	43 (38)	162 (56)	**10.08,*p*** < 0.01**
Energy	310 (77)	86 (77)	224 (78)	0.02, *p* = 0.88

Chi-square test; *p < 0.05; **p ≤ 0.01

**Fig. 2:**
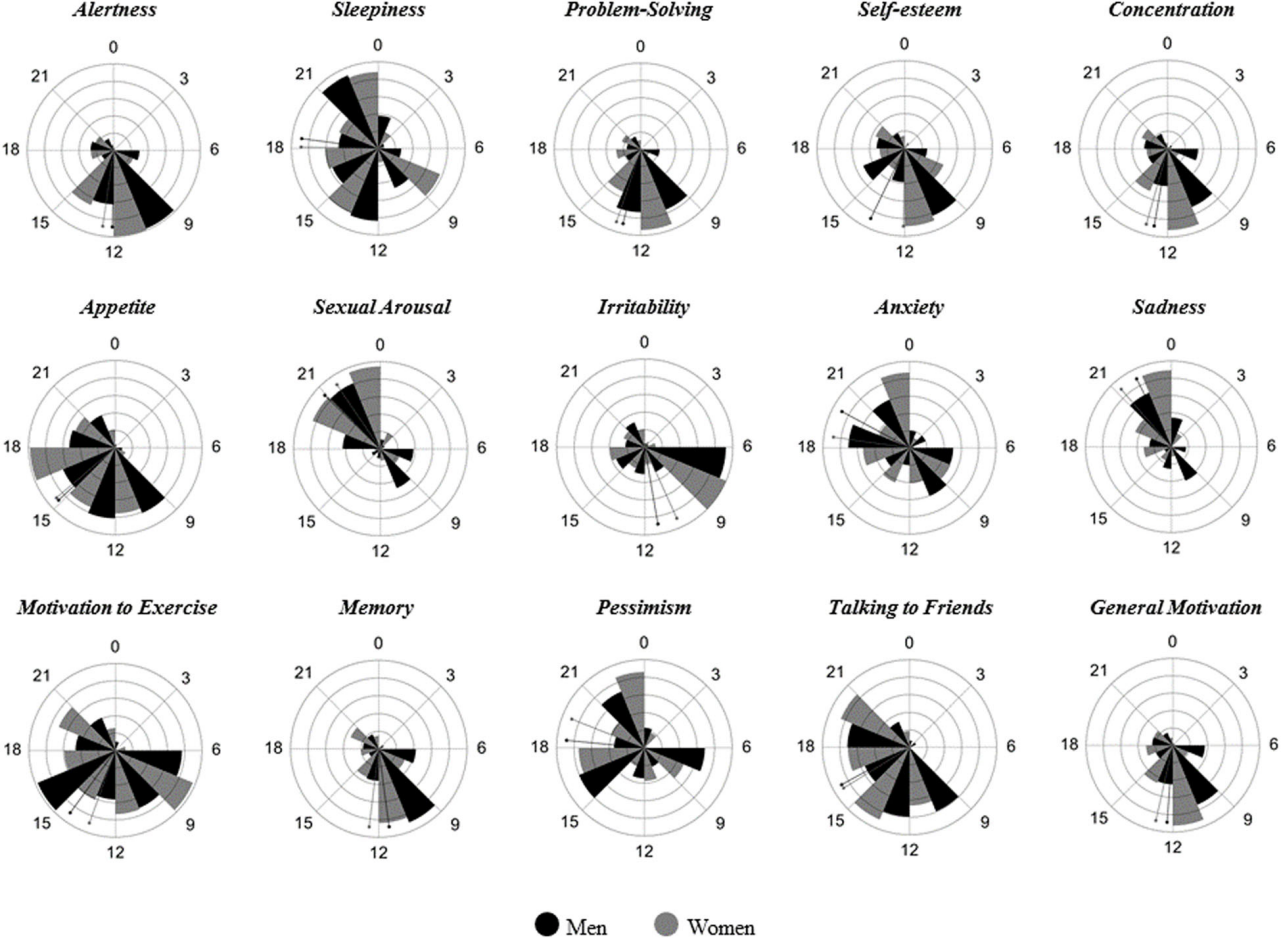
Rose plots for the Mood Rhythm Instrument (MRhI) time variables divided by groups of sex. The circle represents 24 h and each bin represents a 3-h period. The petals magnitudes are based on the proportion of participants of each group presenting a daily peak in the corresponding 3-h period. The bin width is divided equally by the two groups and the petals are laid out sequentially in the bin. The lines represent the circular mean for each group. Men and women do not differ in any MRhI item considering a level of significance of α = 0.05 (Mardia-Watson-Wheeler test for equal distributions)

The comparison of MRhI time variables distribution according to sex is displayed in Rose plots (Fig. 2). The items did not vary between men and women (all *p* > 0.05). Notably, *sleepiness*, *appetite*, *anxiety*, *motivation to exercise*, *pessimism* and *talking to friends* seemed to have a multimodal (i.e. more than one peak time in a 24 h period) pattern of occurrence.

**Fig. 3:**
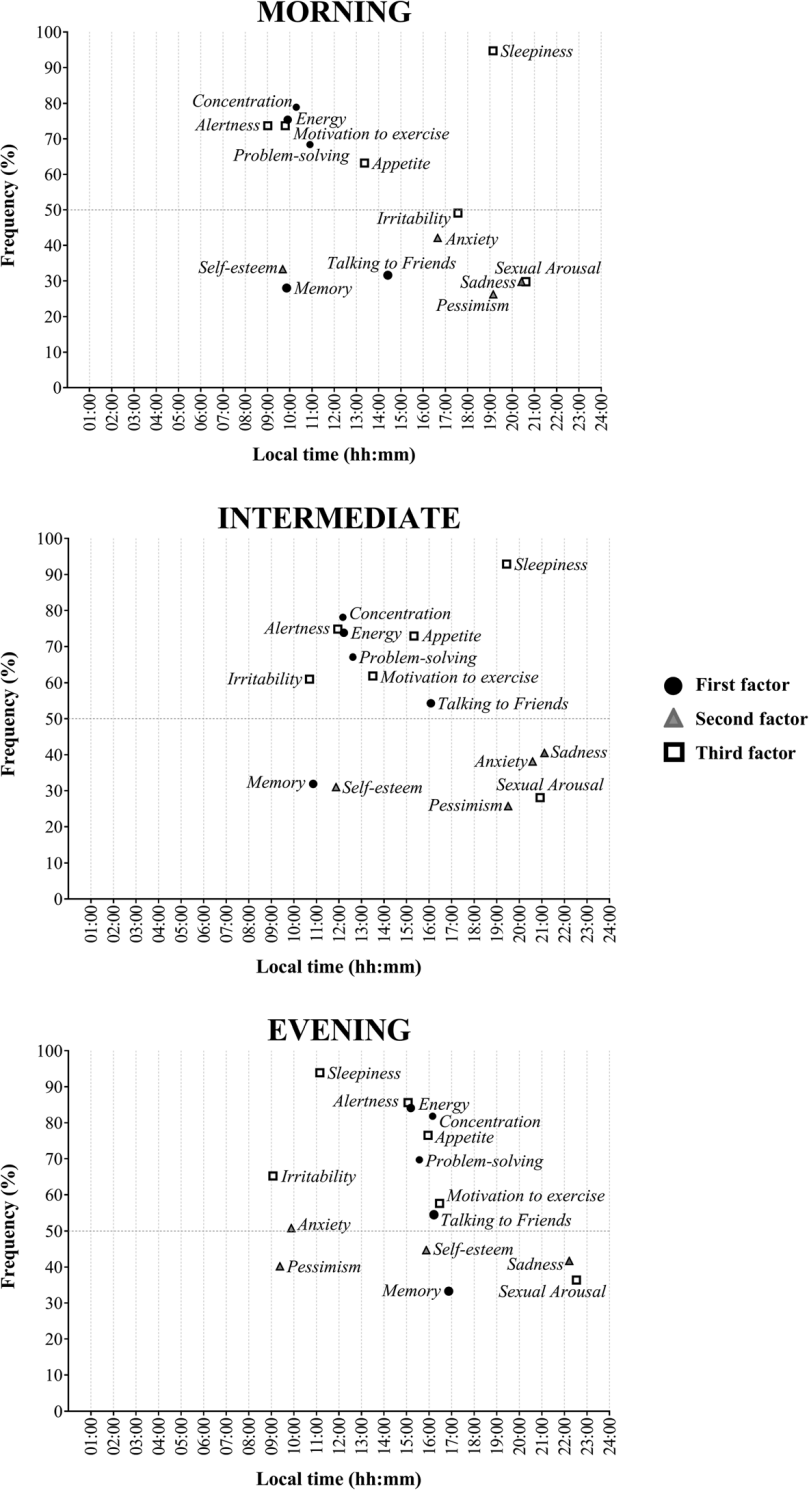
Frequency and peak of each MRhI item. The circular mean of each 24-h peak for mood symptoms is depicted on the x-axis and frequency (%) is depicted on the y-axis

According to linear-circular correlation, all MRhI timing items were significantly correlated with rMEQ scores (Table 3). However, we cannot establish if items are positively or negatively correlated to rMEQ scores due to the nature of a circular measure. Figure 3 presents the frequency of which subjects responded with having a peak for MRhI items and the circular means of the reported peaks according to chronotype. The later the circular means appeared for cognitive and somatic items, the more eveningness the chronotype became (e.g. *alertness, problem-solving, concentration, appetite, motivation to exercise, memory, talking to friends* and *energy*). The opposite occurred with *irritability*, *sleepiness*, *anxiety* and *pessimism*. *Sexual arousal* and *sadness* did not seem to vary among the different chronotypes and showed little rhythmicity.

**Table 3:** Linear-circular correlations between time of peak of MRhI items and rMEQ scores

MRhI items (n)	R-squared	*p*-value
Alertness (309)	0.290	≤0.001
Sleepiness (370)	0.106	≤0.001
Problem-Solving (270)	0.147	≤0.001
Self-esteem (142)	0.206	≤0.001
Concentration (315)	0.247	≤0.001
Appetite (289)	0.028	≤0.001
Sexual Arousal (123)	0.066	≤0.001
Irritability (241)	0.203	≤0.001
Anxiety (170)	0.089	≤0.001
Sadness (157)	0.046	≤0.001
Motivation to Exercise (246)	0.162	≤0.001
Memory (126)	0.231	≤0.001
Pessimism (120)	0.107	≤0.001
Talking to friends (203)	0.020	0.018
Energy (305)	0.221	≤0.001

*Abbreviations*: *rMEQ* Reduced Morningness-Eveningness Questionnaire

## Discussion

The translation of the MRhI to English was adjusted to language and clarity. Importantly, the time scale was modified into the 12-h clock format for a cultural adaptation for the Canadian and most English-speaking countries. The Cronbach’s alpha was 0.75, meaning that the items had an acceptable internal consistency and were adequate, similar to previous validation studies of the MRhI [21, 23].

The three factors solution grouped the items based on the nature of their features. Considering that the first factor explains the greatest percentage of the variance, items in this factor are considered to have an important contribution to the explained variance [43]. It seems that in this Canadian sample, cognitive items are more important in assessing the profile of mood rhythmicity than affective and somatic ones. Yet, in the recent Spanish validation of MRhI, psychometric analysis showed that the first factor grouped somatic items except for *problem-solving* [23].

In line with previous MRhI studies, cognitive and somatic items had more reported peaks than affective items [21, 23]. With the exception of *anxiety*, the affective items exhibited perceived peaks on less than 40% of subjects from the whole sample. When considering sex to compare the frequency of daily peaks, we found significant differences in *irritability*, *anxiety* and *sadness*, which were more frequently reported by women. Notably, these results are consistent with epidemiological data pointing to a higher prevalence of mood and anxiety disorders in women, which is corroborated by biological [44] and socioeconomic [45] contributors. Another factor that may be related to the sex differences observed are alexithymic behaviors, which are more prevalent in men, resulting in a lack of report of negative emotions in this population [46].

Women also reported a higher frequency of daily peaks for *talking to friends*, which is in accordance to a previous British study that evaluated social interaction components of circadian rhythms through phone calls monitoring. Results showed that, comparatively to men, women spent more time on calls with friends in the evening and at night [47]. Interestingly, in terms of sexual arousal, men reported more frequency of sexual arousal peaks than women, which is exactly the opposite from what we observed in Spanish population [23] and also distinct from Brazilians that did not display differences in sexual arousal peak between women and men [21]. Moreover, *problem-solving* and *motivation to exercise* were more frequently pointed as having a daily peak in men. This result can be in part supported by the fact that women are more prone to be extrinsically motivated (with expectations to gain rewards or outcomes) to exercise than intrinsically motivated (aiming personal satisfaction and/or enjoyment), resulting in less motivation for regular physical activity in comparison to men [48]. In contrast to the Brazilian sample, where *alertness* was the only item that differed between sexes, with women reporting more frequently to have a peak than men [21], no differences with regards to *alertness* were found in the Canadian sample.

Overall, we observed that the Canadians reported more sex differences with regards to frequency of perceived peaks than the Brazilian sample. Regarding negative mood and somatic symptomatology, women reported more frequent peaks than men (irritability, anxiety and sadness), while for positive cognitive and somatic activity behaviors men reported more frequent peaks than women (sexual arousal, problem solving and motivation to exercise). As aforementioned, higher prevalence of mood symptoms in women possibly contrasts observations related to the affective items in a sample mostly composed by them. Thus, future research exploring these factors which controls for the relationship between sex and psychiatric symptoms shall bring valuable insights related to the sex differences observed.

The time when items peak did not differ between men and women in any of the MRhI items. Considering that participants could only choose one time peak, even though men reported to have a *sexual arousal* peak in the morning, pointed morning peaks were much more frequent as among women and, therefore, circular means were similar between women and men. A Polish study reported that women had evening peaks of “greatest need for sex”, whereas men had both morning and evening peaks [49]. This multimodal pattern of occurrence was also identified in *appetite* and usually varies among breakfast, lunch and dinner [50]. Subjects also reported peaks of *sleepiness* in the morning, right after midday and at night which is in line with previous analyses of sleepiness expression [51].

As we expected, the circadian typology, measured by means of chronotype, is significantly correlated to all MRhI items time peaks [52]. Participants classified as morning types reported earlier peak times for *concentration*, *alertness*, *problem-solving*, *energy*, *memory*, *motivation to exercise* and *self-esteem*. In contrast, individuals classified as evening types reported that these items peaked later in the day. These results are consistent with previous studies from Europe and United States showing that individuals with morning chronotype performed better in terms of attention, alertness and working memory in the morning and afternoon when compared to individuals with an evening chronotype [53, 54]. Also, depending on the type of problem to solve (e.g. insight or analytic), individuals classified as having a later circadian arousal perform better during later afternoon sessions (between 4 pm and 5:30 pm) [55].

Cognitive performance has been shown to be correlated with individual’s body temperature rhythm. Wright et al. [56] found that cognitive tasks were performed better when body temperature is high and near its circadian peak. Alongside these findings, another study found that individuals with a morning chronotype have earlier temperature rhythms, thus their peaks in cognitive performance such as memory and alertness would also occur earlier than those with evening chronotypes [57]. Our data revealed that individuals with evening chronotype reported earlier peaks of *sleepiness* compared to individuals with morning and intermediate chronotypes. However, it is possible that this finding does not reflect spontaneous behaviors, but rather the consequences of sleep disruption related to diurnal social demands, a hypothesis that is endorsed by the same finding regarding *irritability, pessimism* and *anxiety* (earlier in evening chronotypes). Due to the mean age of 22.2 ± 7 years of our sample, which in general is related to later chronotypes [52], the opposite maladaptation for nocturnal activities could be observed in *talking to friends*. This item peaked at a similar time for intermediate and evening types, and earlier in morning types. Finally, in our sample *sexual arousal, appetite* and *sadness* displayed little variation between chronotypes.

This study has some limitations. We are aware that the MRhI does not reflect mood rhythms independently of external or social factors, as responsibilities and schedules of participants probably influence their responses. However, it is of our interest to evaluate individuals’ self-perception of rhythmicity of mood-related symptoms when inserted in a real-life setting, rather than assessing internal rhythm alone. External factors that exist in a person’s environment are intricate experiences that can also influence how symptoms of mood disorders present themselves. Another limitation is that only one external validation measure was used, albeit it is a well-established questionnaire to evaluate chronotype [30]. Longitudinal monitoring of cognitive, affective, and somatic symptoms using Ecological Momentary Assessment methods should also be considered when validating instruments like the MRhI.

## Conclusions

In conclusion, the results obtained with the English version of the MRhI are consistent with previous chronobiology studies, suggesting that this instrument might be useful to enhance the knowledge of self-perceived daily patterns of mood-related symptoms. The Cronbach’s alpha analysis suggests good internal consistency of this instrument. Cognitive, affective and somatic items presented different frequency of reported peaks and regarding its timing, they seemed to behave accordingly to chronotype. The future directions will be the use of the MRhI instrument in a large sample of individuals with mood disorders, aiming to provide a better understanding of the relationship between daily patterns of mood variability and mental disorders.

## Supplementary information

**Additional file 1.**

## Data Availability

All data generated or analyzed during this study are included in this published article.

## Abbreviations

MRhI: Mood Rhythm Instrument
rMEQ: Reduced Morningness-Eveningness Questionnaire
MRhI-English: English version of the Mood Rhythm Instrument
MAP: Minimum average partial
CD: Comparison Data
CFA: Confirmatory factor analysis
TLI: Tucker-Lewis Index
RMSEA: Root mean square error of approximation

## References

[1] Vos T, Barber RM, Bell B, Bertozzi-Villa A, Biryukov S, Bolliger I (2015). Global, regional, and national incidence, prevalence, and years lived with disability for 301 acute and chronic diseases and injuries in 188 countries, 1990-2013: A systematic analysis for the Global Burden of Disease Study 2013. Lancet.

[2] Pittenger C, Duman RS (2008). Stress, depression, and neuroplasticity: a convergence of mechanisms. Neuropsychopharmacology.

[3] Krishnan V, Nestler EJ (2010). Linking molecules to mood: new insight into the biology of depression. Am J Psychiatry.

[4] Angst J, Azorin JM, Bowden CL, Perugi G, Vieta E, Gamma A, Young AH, BRIDGE Study Group (2011). Prevalence and characteristics of undiagnosed bipolar disorders in patients with a major depressive episode: the BRIDGE study. Arch Gen Psychiatry.

[5] Shefer G, Henderson C, Howard LM, Murray J, Thornicroft G (2014). Diagnostic overshadowing and other challenges involved in the diagnostic process of patients with mental illness who present in emergency departments with physical symptoms--a qualitative study. PLoS One.

[6] McClung CA (2013). How might circadian rhythms control mood? Let me count the ways. Biol Psychiatry.

[7] Slyepchenko A, Allega OR, Leng X, Minuzzi L, Eltayebani MM, Skelly M, Sassi RB, Soares CN, Kennedy SH, Frey BN (2019). Association of functioning and quality of life with objective and subjective measures of sleep and biological rhythms in major depressive and bipolar disorder. Aust N Z J Psychiatry.

[8] Ávila Moraes C, Cambras T, Diez-Noguera A, Schimitt R, Dantas G, Levandovski R, Hidalgo MP (2013). A new chronobiological approach to discriminate between acute and chronic depression using peripheral temperature, rest-activity, and light exposure parameters. BMC Psychiatry.

[9] Walker WH, Walton JC, DeVries AC (2020). Circadian rhythm disruption and mental health. Transl Psychiatry.

[10] Charrier A, Olliac B, Roubertoux P, Tordjman S (2017). Clock genes and altered sleep–wake rhythms: Their role in the development of psychiatric disorders. Int J Mol Sci.

[11] Geoffroy PA, Micoulaud Franchi JA, Lopez R, Poirot I, Brion A, Royant-Parola S (2017). Comment caractériser et traiter les plaintes de sommeil dans les troubles bipolaires ?. Encephale.

[12] Hertenstein E, Feige B, Gmeiner T, Kienzler C, Spiegelhalder K, Johann A (2019). Insomnia as a predictor of mental disorders: A systematic review and meta-analysis. Sleep Med Rev.

[13] Boivin DB (2000). Influence of sleep-wake and circadian rhythm disturbances in psychiatric disorders. J Psychiatry Neurosci.

[14] Proudfoot J, Whitton A, Parker G, Doran J, Manicavasagar V, Delmas K (2012). Triggers of mania and depression in young adults with bipolar disorder. J Affect Disord.

[15] Tonon AC, Fuchs DFP, Barbosa Gomes W, Levandovski R, Pio de Almeida Fleck M, MPL H (2017). Nocturnal motor activity and light exposure: Objective actigraphy-based marks of melancholic and non-melancholic depressive disorder. Brief report. Psychiatry Res.

[16] Krane-Gartiser K, Vaaler AE, Fasmer OB, Sørensen K, Morken G, Scott J (2017). Variability of activity patterns across mood disorders and time of day. BMC Psychiatry.

[17] McGowan NM, Goodwin GM, Bilderbeck AC, Saunders KEA. Actigraphic patterns, impulsivity and mood instability in bipolar disorder, borderline personality disorder and healthy controls. Acta Psychiatr Scand. 2020. 10.1111/acps.13148 [Epub ahead of print] PubMed PMID: 31916240. 10.1111/acps.13148 PMC7216871 31916240

[18] Bottai T, Biloa-Tang M, Christophe S, Dupuy C, Jacquesy L, Kochman F (2010). Thérapie interpersonnelle et aménagement des rythmes sociaux (TIPARS) : du concept anglo-saxon l’expérience franaise. Encephale.

[19] Haynes PL, Gengler D, Kelly M (2016). Social Rhythm Therapies for Mood Disorders: an Update. Curr Psychiatry Rep.

[20] Pail G, Huf W, Pjrek E, Winkler D, Willeit M, Praschak-Rieder N (2011). Bright-light therapy in the treatment of mood disorders. Neuropsychobiology.

[21] De Souza CM, Carissimi A, Costa D, Francisco AP, Medeiros MS, Ilgenfritz CA (2016). The mood rhythm instrument: Development and preliminary report. Rev Bras Psiquiatr.

[22] Francisco AP, de Oliveira MAB, Carissimi A, Fabris RC, Ilgenfritz CAV, de Souza CM (2017). Spanish Translation of the Mood Rhythm Instrument: a Novel Approach To Mood Evaluation. Clin Biomed Res.

[23] Carissimi A, Oliveira MAB, Frey BN, Navarro JF, Hidalgo MP, Adan A. Validation and psychometric properties of the Spanish mood rhythm instrument. Biol Rhythm Res. 2019. 10.1080/09291016.2019.1675023.

[24] Pilz LK, Carissimi A, Oliveira MAB, Francisco AP, Fabris RC, Medeiros MS (2018). Rhythmicity of Mood Symptoms in Individuals at Risk for Psychiatric Disorders. Sci Rep.

[25] Pilz LK, Carissimi A, Francisco AP, Oliveira MAB, Slyepchenko A, Epifano K, et al. Prospective assessment of daily patterns of mood-related symptoms. Front Psychiatry. 2018;9 (AUG). Available from: https://www.frontiersin.org/article/10.3389/fpsyt.2018.00370/full. [cited 2019 May 21]. 10.3389/fpsyt.2018.00370 PMC6110875 30186188

[26] Mistlberger RE, Skene DJ (2004). Social influences on mammalian circadian rhythms: animal and human studies. Biol Rev Camb Philos Soc.

[27] Gradisar M, Gardner G, Dohnt H (2011). Recent worldwide sleep patterns and problems during adolescence: A review and meta-analysis of age, region, and sleep. Sleep Med.

[28] Soldatos CR, Allaert FA, Ohta T, Dikeos DG (2005). How do individuals sleep around the world? Results from a single-day survey in ten countries. Sleep Med.

[29] Smith MR, Burgess HJ, Fogg LF, Eastman CI (2009). Racial differences in the human endogenous circadian period. Yamazaki S, editor. PLoS One.

[30] Adan A, Almirall H (1991). Horne & Östberg morningness-eveningness questionnaire: A reduced scale. Pers Individ Dif.

[31] Danielsson K, Sakarya A, Jansson-Fröjmark M (2019). The reduced Morningness-Eveningness questionnaire: psychometric properties and related factors in a young Swedish population. Chronobiol Int.

[32] Tavakol M, Dennick R (2011). Making sense of Cronbach’s alpha. Int J Med Educ.

[33] Bartholomew DJ, Steele F, Moustaki I, Galbraith J (2008). Analysis of Multivariate Social Science Data, Second Edition. Int Stat Rev.

[34] Mislevy R (1986). Recent developments in the factor analysis of categorical variables. J Educ Stat.

[35] Bock RD, Gibbons R, Muraki E (1988). Full-information item factor analysis. Appl Psychol Meas.

[36] Finch WH (2011). A Comparison of Factor Rotation Methods for Dichotomous Data. J Mod Appl Stat Methods.

[37] Velicer WF (1976). Determining the number of components from the matrix of partial correlations. Psychometrika.

[38] Horn JL (1965). A rationale and test for the number of factors in factor analysis. Psychometrika.

[39] Ruscio J, Roche B (2012). Determining the number of factors to retain in an exploratory factor analysis using comparison data of known factorial structure. Psychol Assess.

[40] Brown TA. Methodology in the social sciences. Confirmatory factor analysis for applied research. 2nd ed. New York: The Guilford press; 2015.

[41] Mardia KV, Jupp PE (1999). Directional Statistics.

[42] Mardia KV. Statistics of directional data. J R Stat Soc Ser B Methodol. 1975;37:349–93.

[43] Nias DKB (2003). The handbook of psychological testing. Vol. 20, personality and individual differences.

[44] Kuehner C (2017). Why is depression more common among women than among men?. Lancet Psychiatry.

[45] Lim GY, Tam WW, Lu Y, Ho CS, Zhang MW, Ho RC (2018). Prevalence of Depression in the Community from 30 Countries between 1994 and 2014 /692/699/476/1414 /692/499 article. Sci Rep.

[46] Sullivan L, Camic PM, JSL B (2015). Masculinity, alexithymia, and fear of intimacy as predictors of UK men’s attitudes towards seeking professional psychological help. Br J Health Psychol.

[47] Aledavood T, López E, Roberts SGB, Reed-Tsochas F, Moro E, Dunbar RIM (2015). Daily rhythms in mobile telephone communication. Lambiotte R, editor. PLoS One.

[48] Ryan RM (1997). Intrinsic motivation and exercise adherence. Int J Sport Psychol.

[49] Jankowski KS, Díaz-Morales JF, Randler C (2014). Chronotype, gender, and time for sex. Chronobiol Int.

[50] Scheer FAJL, Morris CJ, Shea SA (2013). The internal circadian clock increases hunger and appetite in the evening independent of food intake and other behaviors. Obesity.

[51] Campbell SS, Murphy PJ (2007). The nature of spontaneous sleep across adulthood. J Sleep Res.

[52] Adan A, Archer SN, Hidalgo MP, Di Milia L, Natale V, Randler C (2012). Circadian typology: A comprehensive review. Chronobiol Int.

[53] Matchock RL, Toby Mordkoff J (2009). Chronotype and time-of-day influences on the alerting, orienting, and executive components of attention. Exp Brain Res.

[54] Schmidt C, Collette F, Reichert CF, Maire M, Vandewalle G, Peigneux P (2015). Pushing the limits: Chronotype and time of day modulate working memory-dependent cerebral activity. Front Neurol.

[55] Wieth MB, Zacks RT (2011). Time of day effects on problem solving: when the non-optimal is optimal. Think Reason.

[56] Wright KP, Hull JT, Czeisler CA (2015). Relationship between alertness, performance, and body temperature in humans. Am J Physiol Integr Comp Physiol.

[57] Lack L, Bailey M, Lovato N, Wright H (2009). Chronotype differences in circadian rhythms of temperature, melatonin, and sleepiness as measured in a modified constant routine protocol. Nat Sci Sleep.

