# Supplementary material for: Validation of the English version of the Mood Rhythm Instrument
Source: BMC Psychol. 2020 Apr 17;8:35. doi: 10.1186/s40359-020-00397-2 (PMC7165411; doi:10.1186/s40359-020-00397-2)
Supplement: Supplementary file 1 — Additional file 1. [file 40359_2020_397_MOESM1_ESM.pdf]

Supplemental material for **English Version of Mood Rhythm Instrument**

Mood Rhythm Instrument - MRI

Date: ____/_____/______ Sex: ( ) F ( ) M Subject ID: ________________

Date of last period: ____/____/_____

Age: _______ Level of education (years of schooling): _______

Please read carefully the following examples before answering the questions:

Following each question, a 24-hour period is shown. Each number represents an hour of the clock. The line between the numbers represents the minutes.

Example (A): if you mark a line between 7AM and 8AM, it will represent 7:30 AM

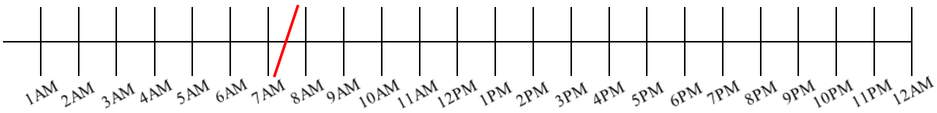

(B) If you mark a line on 3PM, it will represent 3:00 PM

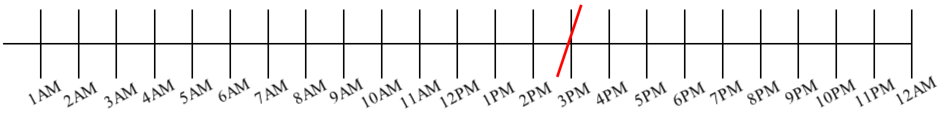

Answer the following questions according to the **last 15 days**, taking into account how you have felt most of the time, on the majority of the days.

1. Is there a specific time of the day when you have felt more alert?
( ) Yes ( ) No
If you answer yes, indicate below the approximate hour:

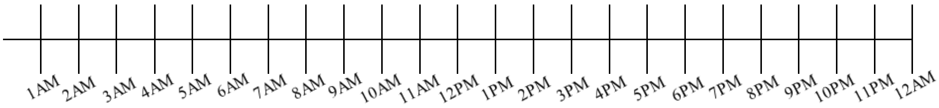

2. Is there a specific time of the day when you have felt sleepier?
( ) Yes ( ) No
If you answer yes, indicate below the approximate hour:

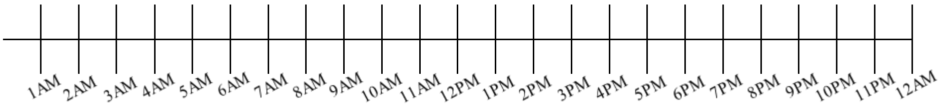

3. Is there a specific time of the day when you have felt more capable of solving daily problems?
( ) Yes ( ) No
If you answer yes, indicate below the approximate hour:

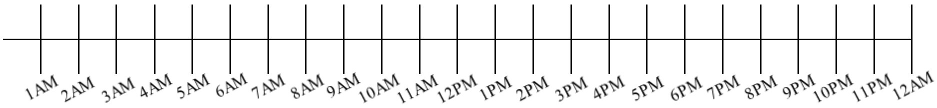

4. Is there a specific time of the day when your self-esteem has been higher?
( ) Yes ( ) No
If you answer yes, indicate below the approximate hour:

1AM 2AM 3AM 4AM 5AM 6AM 7AM 8AM 9AM 10AM 11AM 12PM 1PM 2PM 3PM 4PM 5PM 6PM 7PM 8PM 9PM 10PM 11PM 12AM

5. Is there a specific time of the day when you been able to concentrate better?
( ) Yes ( ) No
If you answer yes, indicate below the approximate hour:

1AM 2AM 3AM 4AM 5AM 6AM 7AM 8AM 9AM 10AM 11AM 12PM 1PM 2PM 3PM 4PM 5PM 6PM 7PM 8PM 9PM 10PM 11PM 12AM

6. Is there a specific time of the day when you have had an increased appetite?
( ) Yes ( ) No
If you answer yes, indicate below the approximate hour:

1AM 2AM 3AM 4AM 5AM 6AM 7AM 8AM 9AM 10AM 11AM 12PM 1PM 2PM 3PM 4PM 5PM 6PM 7PM 8PM 9PM 10PM 11PM 12AM

7. Is there a specific time of the day when your libido (sexual arousal) has been higher?
( ) Yes ( ) No
If you answer yes, indicate below the approximate hour:

1AM 2AM 3AM 4AM 5AM 6AM 7AM 8AM 9AM 10AM 11AM 12PM 1PM 2PM 3PM 4PM 5PM 6PM 7PM 8PM 9PM 10PM 11PM 12AM

8. Is there a specific time of the day when you have felt more irritable?
( ) Yes ( ) No
If you answer yes, indicate below the approximate hour:

1AM 2AM 3AM 4AM 5AM 6AM 7AM 8AM 9AM 10AM 11AM 12PM 1PM 2PM 3PM 4PM 5PM 6PM 7PM 8PM 9PM 10PM 11PM 12AM

9. Is there a specific time of the day when you have felt more anxious?
( ) Yes ( ) No
If you answer yes, indicate below the approximate hour:

1AM 2AM 3AM 4AM 5AM 6AM 7AM 8AM 9AM 10AM 11AM 12PM 1PM 2PM 3PM 4PM 5PM 6PM 7PM 8PM 9PM 10PM 11PM 12AM

10. Is there a specific time of the day when you have felt more sad?
( ) Yes ( ) No
If you answer yes, indicate below the approximate hour:

1AM 2AM 3AM 4AM 5AM 6AM 7AM 8AM 9AM 10AM 11AM 12PM 1PM 2PM 3PM 4PM 5PM 6PM 7PM 8PM 9PM 10PM 11PM 12AM

11. Is there a specific time of the day when you have felt more motivated to exercise?
( ) Yes ( ) No
If you answer yes, indicate below the approximate hour:

1AM 2AM 3AM 4AM 5AM 6AM 7AM 8AM 9AM 10AM 11AM 12PM 1PM 2PM 3PM 4PM 5PM 6PM 7PM 8PM 9PM 10PM 11PM 12AM

12. Is there a specific time of the day when your memory has been better?
( ) Yes ( ) No
If you answer yes, indicate below the approximate hour:

1AM 2AM 3AM 4AM 5AM 6AM 7AM 8AM 9AM 10AM 11AM 12PM 1PM 2PM 3PM 4PM 5PM 6PM 7PM 8PM 9PM 10PM 11PM 12AM

13 Is there a specific time of the day when you have been more pessimistic?
( ) yes ( ) No
If you answer yes, indicate below the approximate hour:

1AM 2AM 3AM 4AM 5AM 6AM 7AM 8AM 9AM 10AM 11AM 12PM 1PM 2PM 3PM 4PM 5PM 6PM 7PM 8PM 9PM 10PM 11PM 12AM

14. Is there a specific time of the day when you have preferred talking to friends face-to-face?
( ) yes ( ) No
If you answer yes, indicate below the approximate hour:

1AM 2AM 3AM 4AM 5AM 6AM 7AM 8AM 9AM 10AM 11AM 12PM 1PM 2PM 3PM 4PM 5PM 6PM 7PM 8PM 9PM 10PM 11PM 12AM

15. Is there a specific time of day when you have had more energy and motivation to do things?
( ) yes ( ) No
If you answer yes, indicate below the approximate hour:

1AM 2AM 3AM 4AM 5AM 6AM 7AM 8AM 9AM 10AM 11AM 12PM 1PM 2PM 3PM 4PM 5PM 6PM 7PM 8PM 9PM 10PM 11PM 12AM
